# Trocar site post incisional hernia: about 19 cases

**DOI:** 10.11604/pamj.2018.29.183.14467

**Published:** 2018-03-28

**Authors:** Karim Nacef, Mohamed Ali Chaouch, Asma Chaouch, Mohamed Ben Khalifa, Mossaab Ghannouchi, Moez Boudokhane

**Affiliations:** 1 Faculty of Medicine of Monastir, Department of General Surgery, Taher Sfar Hospital, Mahdia, Tunisia

**Keywords:** Port-site incisional hernia, pneumoperitoneum, port closure, incisional hernia, laparoscopy

## Abstract

It is commonly admitted that laparoscopic surgery has the advantage of abdominal wall preservation. Therefore, having port-site incisional hernia caused by trocars of laparoscopy must be avoided. The aim of this work is to specify predictive factors, therapeutic modalities and to insist on prevention of this avoidable complication. It is a retrospective and descriptive study over a period of 10 years, between January 2006 and December 2015. This series includes 19 consecutive patients who present port-site incisional hernia. Age, initial intervention, site and size of the trocars incisional hernia, diagnostic method, delay and type of the second procedure with the final results were examined and recorded. Our study contains 19 female. The average age was 55 years (29-78). Risk factors were resent in 12 patients. All our patients were operated initially by laparoscopic approach. The average onset time was 6.6 months (3-12). Fourteen patients presented swelling at the trocar site and 5 patients had an emergent surgery due to the strangulation of the port-site incisional hernia. For these five patients a primary suture was made. Hernia content was the great omentum in 11 cases and small bowel in 8 cases. It was umbilical in 16 patients and in the left flank in 3 patients. They occur all where it was placed a 10 mm trocar. The evolution was suitable in all cases. There were two recurrences, one after primary suture and the other after a mesh repair. Port-site incisional hernia is rare. The most incriminated risk factors are essentially trocar size, obesity and open coelioscopy. Vital prognosis can be engaged if port-site incisional hernia is incarcerated or strangulated then prevention is necessary.

## Introduction

It is commonly admitted that laparoscopic surgery has the advantage of abdominal wall preservation. Therefore, having port-site incisional hernia (PSIH) caused by trocars of laparoscopy must be avoided. This complication is rare, it presents 1% of laparoscopic surgeries [1]. It can occur immediately or late postoperatively. Treatment of this complication can be done by suture or mesh repair. But the best treatment remains prevention. This prevention requires knowledge of the risk factors for this condition. The aim of this work is to specify predictive factors, therapeutic modalities and to insist on prevention of this avoidable complication.

## Methods

It is a retrospective and descriptive study over a period of 10 years, between January 2006 and December 2015. This series includes 19 consecutive patients who present PSIH. These patients were operated initially in two surgical departments: department of general surgery and department of gynecology. These cases were examined and recorded elements were respectively: age, initial intervention, site and size of the trocars incisional hernia, diagnostic method, delay and type of the second procedure with the final results. No attempt was made to define the total number of operative laparoscopies performed in these two participating departments during the study period.

## Results

Our study contains 19 female. The different results are shown in Table 1. The average age was 55 years with extremes from 29 to 78 years. Risk factors were present in 12 patients. All our patients were operated initially by laparoscopic approach. Fifteen operated in general surgery department and four in gynecological department. The onset time varies between 03 and 12 months with an average of 6.6 months. Regarding the circumstances of discovery, 14 patients presented swelling at the trocar site and 5 were admitted and operated urgently due to strangulation of the PSIH. The surgical treatment consists of a suture or a mesh repair. For the patients operated in emergency, a suture was made because the contents was small bowel associated to a ladle effusion in the hernia sac. The content was the great omentum in 11 cases and small bowel in 8 cases. Any digestive resection was needed. Post incisional hernia is located at the umbilical opening in 16 patients and in the left flank in 3 patients. They occur all where it was placed a 10 mm trocar. No incisional hernias was reported of 5 mm trocar port has been reported in our series. The evolution was suitable in all cases. There were two recurrences, one after suture and the other after mesh repair.

**Table 1:** Different characteristics of ours patients

Age	Risk factors	Indication	Site	Delay (months)	Emergent operation	Repair	Recurrence
48	Hypothyroidism	TL	Umbilical	-	Yes	Suture	-
63	Asthma/Obesity	CA	Umbilical	-	No	Suture	-
70	Obesity	GORD	Left flank	-	No	Mesh-R	-
42	-	CC	Umbilical	-	No	Mesh-R	-
74	Diabetes	CA	Umbilical	-	No	Mesh-R	-
34	-	CC	Umbilical	-	No	Mesh-R	-
69	-	CC	Umbilical	04	No	Mesh-R	-
54	-	CC	Umbilical	-	Yes	Suture	-
47	-	CC	Umbilical	-	No	Mesh-R	-
56	Constipation / HL	EP	Umbilical	-	No	Mesh-R	-
43	-	CA	Umbilical	10	No	Mesh-R	+ 90 jrs
76	Obesity	CC	Umbilical	-	Yes	Suture	-
56	Smoking / HL	CC+UH	Umbilical	04	Yes	Suture	+ 120 jrs
41	-	OC	Left flank	12	No	Mesh-R	-
78	-	CC	Umbilical	-	No	Mesh-R	-
29	-	CC	Umbilical	03	No	Mesh-R	-
56		CC	Umbilical	07	No	Mesh-R	-
70	Diabetes / obesity	CC	Left flank	-	Yes	Mesh-R	-
55	-	TL	Umbilical	-	No	Mesh-R	-

**CC:** chronic choecystis; **TL:** tubal ligation; **EP:** ectopic pregnancy; **HL:** heavy lifting; **UH:** umbilical hernia; **GORD:** gastroesophageal reflux disease; **Mesh-R:** mesh repair

## Discussion

The incidence of this complication is underestimated because many patients consult only when they are symptomatic. Then the real incidence can be established only if an abdominal CT-scan will be done for each patient operated with a laparoscopic approach. The incidence is about 1%, Leibl et al. in a comparative study relieve an incidence between 0.8% and 1.2% [1]. This complication is associated with many risk factors such as umbilical trocar position, trocar size ≥ 10 mm, advanced age, obesity and malnutrition [2,3]. Several studies prove that trocar size and type are the major factors predisposing the development of PSIH. Then, it is necessary to favor non-conical shape trocars and those inferior to 10 mm [1,4,5] because for the same trocar location increasing trocar size from 10 to 12 mm the risk of PSIH rise up from 0.23% to 3.1% [6]. Kadar et al. found that open laparoscopy is associated with a higher rate of PSIH then Veress needle [6]. For the trocar localization, the results of different studies are divergent. Some describe only extra-umbilical PSIH [6], only umbilical PSIH [7] or extra-umbilical and umbilical PSIH with different proportions [5,8]. But, Loriau et al. in a systematic review retained the percentage of 31% of umbilical PSIH localization [9]. Several other factors may influence the risk of this complication such as iterative manipulations, prolonged interventions, repeated repositioning and use of a trocar anchoring system but they have never been studied. Treatment remains surgical and it can be by classic or laparoscopic approach [8]. Most published patients were classically treated. A Randomized multicenter clinical trial comparing classic and laparoscopic PSIH treatment of 194 Cases [10]. For patient treated with the laparoscopic approach, median loss of blood during operation was significantly lower as well as the use of drainage. While the operating time was longer. Perioperative complications were significantly higher after laparoscopy (9% vs. 2%) but recurrence was lower than laparotomy (14% vs 18%). PSIH can occur either in immediate postoperative or later [5]. For surgeons who do not close the trocar incision, Hitoshi et al. reclaim that 14 days after surgery to be the turning point for decision making. Before this delay, a surgical suture is necessary behind the risk of small bowel obstruction. Late-onset, surgical repair procedure for PSIH needs to be established and a larger series study is needed [3]. In the literature, there is no study that compares results according to the different types of meshes. Prevention is necessary for patients with risk factors. It goes through closing trocars port incision. Some authors recommend to close incisions of trocar 5 mm [11], another of 8 mm [8] but the majority of authors recommend closing trocars incision > 10 mm [2]. This is not enough to prevent this complication. It is pre and intraoperative. Systematic examination of the umbilicus allows to explore abdominal cavity and to treat associated umbilical hernia [7]. Intra-operative total pneumoperitoneum exsufflation, total patient relaxation until the closure of skin and avoid heavy lifting for at least one month postoperatively can reduce the risk of this complication [12].

## Conclusion

PSIH is rare. The most incriminated risk factors are essentially trocar size, obesity and open coelioscopy. Vital prognosis can be engaged if PSIH is incarcerated. Prevention is necessary. This prevention is based essentially on the closure of trocar orifices greater than 10 mm.

### What is known about this topic

- This is a rare complication of laparoscopic surgery;
- If complicated, an emergent surgery is required;
- Treatment is surgical.

### What this study adds

- How to prevent this uncommon complication;
- General characteristics (epidemiology, clinical, radiological, treatment) of this complication.

## Competing interests

The authors declare no conflict of interests.
